# Aetiologies of pericardial effusion among medical patients at Nelson Mandela Academic Hospital, Mthatha, South Africa: A cross-sectional study

**DOI:** 10.4102/jcmsa.v4i1.401

**Published:** 2026-07-20

**Authors:** Thandazile O. Fathuse, Guillermo A. Pulido Estrada

**Affiliations:** 1 Department of Internal Medicine, Nelson Mandela Academic Hospital, Mthatha, South Africa; 2 Department of Internal Medicine, Faculty of Medicine and Health Sciences, Walter Sisulu University, Mthatha, South Africa; 3 School of Public Health, Faculty of Medicine and Health Sciences, Walter Sisulu University, Mthatha, South Africa

**Keywords:** pericardial effusion, echocardiography, tuberculosis, Mthatha, HIV, developing country, resource constraints

## Abstract

**Background:**

Pericardial effusion aetiology varies across geographical locations and epidemiological contexts. In the developing world, tuberculosis (TB), catalysed by human immunodeficiency virus (HIV), is the leading aetiology, while in the developed world, it is idiopathic. The TB epidemic and paucity of recent, regional context aetiological profiling may overshadow other aetiologies in the empirical treatment guidelines.

**Methods:**

A retrospective cross-sectional study of clinical records was conducted to investigate the aetiologies of pericardial effusion in adult patients at Nelson Mandela Academic Hospital (NMAH) in Mthatha, South Africa, between January 2022 and October 2025.

**Results:**

A total of 141 patients were included in this study, with females constituting 52.5% and males (47.5%). The most common presentation symptom was dyspnoea (97.9%), followed by fatigue (84.4%). The most common signs on examination were muffled heart sounds (92.2%) and increased jugular venous pressure (JVP) (61.0%). Idiopathic pericardial effusion was the most common aetiology (29.8%), followed closely by TB (29.1%) and malignancy (9.9%). Among HIV-positive patients, TB was the predominant cause (55.6%), followed by HIV-related pericardial effusion (25.9%). In contrast, HIV-negative patients most commonly had idiopathic pericardial effusion (35.1%), followed by TB (22.8%) and malignant causes (11.4%).

**Conclusion:**

Pericardial effusion in this setting has a heterogeneous aetiological profile and therefore calls for a systematic diagnostic approach to improve aetiological diagnosis and guide appropriate management in high TB and HIV prevalence settings.

**Contribution:**

The addition to the limited regional data on the aetiologies of pericardial effusion in a resource-limited South African setting will inform appropriate diagnostic prioritisation, empiric management strategies and resource allocation in similar settings.

## Introduction

Pericardial effusion, defined as an excessive accumulation of fluid in the pericardial sac, is a potentially life-threatening cardiac condition.^1,2^ Due to various insults to the heart and its pericardium, fluid between the two layers covering the heart may accumulate, exceeding the normal 15 mL – 50 mL volume, leading to a pericardial effusion. When a patient experiences this phenomenon, they may present with acute symptoms requiring emergency care, or it may just be an incidental finding during an echocardiographic examination.^2,3,4^ Without prompt treatment, the fluid may compress the heart, leading to cardiac tamponade and possibly death.^2,5^

It is the accumulated fluid volume and the rate at which pressure builds up in the sac that determine the symptom manifestation of pericardial effusion, whether life-threatening cardiac tamponade or benign.^1,6^ The most common presentation symptoms and signs of pericardial effusion include orthopnoea, dyspnoea with exercise, chest pain, fever and cough.^2,7^ In cardiac tamponade, the heart compression may cause the patient tachycardia, elevated jugular venous pressure (JVP), muffled and distant heart sounds, hypotension and pulsus paradoxus on examination.^1,2,3,8^

The incidence and prevalence of pericardial effusion are 3% and 5% – 7%, respectively,^6^ with much of the data from developed rather than developing countries.^6,9^ However, the epidemiological profile varies by the geographical location of the population, setting, developmental status, disease burden status of the region and the diagnostic criteria used.^4,5,9^ The variable aetiology can be classified as inflammatory and non-inflammatory, acute and chronic.^3,5,6^

Developed countries report idiopathic causes, followed by viral infections, malignancies, iatrogenic causes, auto-inflammatory causes and the recent coronavirus disease 2019 (COVID-19).^1,6^ In contrast, developing countries report tuberculosis (TB) as the leading cause of pericardial effusion, with a prevalence of about 60%, followed by idiopathic, uraemic and malignancy.^1,5^ The TB scourge in sub-Saharan Africa has been perpetuated further by the human immunodeficiency virus (HIV) epidemic, as it increases susceptibility to extrapulmonary TB.^1,10^ When comparing pericardial cases caused by TB between African and non-African cases, there is a significant difference in prevalence (69.5% vs <4%).^11^

The HIV and TB syndemic, along with the resource challenges that limit the investigative capacity to make a definite diagnosis in developing settings, often creates a tendency to presume associated pericardial effusion with TB and to initiate TB therapy empirically.^12^ This is further exacerbated by the paucity of aetiological studies in TB in developing countries.^6,9^ However, there appears to be a growing number of case studies indicating that certain causes of pericardial effusion in South Africa are often overlooked. This oversight is largely due to the strong emphasis in the literature on TB, which can lead to cases being misdiagnosed as TB. Consequently, alternative diagnoses are typically considered only after the initial treatment for presumed TB has failed.^13^

Nelson Mandela Academic Hospital (NMAH), situated in Mthatha in the Eastern Cape province, serves a largely rural and socioeconomically disadvantaged population disproportionately affected by infectious diseases such as TB and HIV. In this setting, the paucity of published data specifically categorising the local aetiological patterns of pericardial effusion leads clinicians to rely on general statistics, which may not accurately reflect the disease burden and healthcare challenges faced by the NMAH patient population, thereby delaying definitive diagnosis and management. More research is therefore needed to understand and potentially uncover the aetiological profile of pericardial effusion in Mthatha, South Africa. Determining the aetiological profile in this study’s context will enhance the accuracy of diagnostic and therapeutic interventions, providing effective management guidelines specific to the local clinical context.

This study, therefore, aimed to determine the aetiologies of pericardial effusion and the associated demographic and clinical characteristics among medical patients at NMAH, Mthatha, South Africa. By providing local epidemiological data, this study seeks to inform context-appropriate clinical decision-making and to contribute to a broader understanding of pericardial disease patterns in high-TB and HIV-burden settings.

## Research methods and design

### Study design

This was a retrospective cross-sectional study design that involved a review of hospital records to determine the aetiologies of pericardial effusion among medical patients at NMAH, Mthatha, South Africa, between January 2022 and October 2025.

### Setting

This study was conducted at NMAH, a tertiary referral hospital located in central Mthatha, Eastern Cape, South Africa. Nelson Mandela Academic Hospital receives referrals from regional and district hospitals in the OR Tambo and Alfred Ndzo district municipalities, as well as from surrounding areas, for tertiary-level healthcare. The population served by NMAH consists mainly of underserved rural–urban black communities with a catchment population of approximately 3.5 million that face a high burden of diseases, including a significant prevalence of TB and HIV.^14^

The hospital has specialised departments, including emergency medicine, paediatrics, obstetrics and gynaecology, surgery, radiology and internal medicine. The internal medicine department has 120 beds and operates through various firms or units. The cardiology unit routinely cares for patients aged 13 years and older with cardiovascular conditions. This unit is equipped with diagnostic tools, including echocardiography, which is performed only by trained physicians.

### Study population and sampling strategy

The study population included all clinical records of patients aged 13 years and older who were diagnosed with pericardial effusion by echocardiography and underwent pericardiocentesis at NMAH, Department of Internal Medicine between January 2022 and October 2025. Surgical-related or post-traumatic causes of pericardial effusion were excluded, and pregnant women were also excluded from this study. Other medical records were excluded due to incompleteness, missing laboratory investigations and missing pericardial fluid analysis (some records were excluded because of non-drainable effusion), thus yielding insufficient diagnostic information to determine aetiologies. A majority of this cohort was presumably diagnosed with TB pericardial effusion and on TB empirical therapy during the study period, including one with confirmed constrictive pericarditis, but had no drainable effusion. All available and eligible patient records during the study period were included through consecutive sampling.

### Sample size

The sample size was not calculated in this study. All records retrievable during the period were included.

### Data collection

For this study, data were collected using a structured data extraction tool in Microsoft Excel (Microsoft 365, Version 16) that was specifically designed for this study. The data extraction tool was designed based on the literature, clinical variables commonly recorded in clinical records of patients with pericardial effusion, the diagnostic modalities and laboratory investigations, and the study’s objectives. The key variables that informed this tool were patient demographics, including patient code, age, gender, date of presentation and discharge date, clinical presentation including signs and symptoms, vital signs, HIV status (date of diagnosis, CD4 count, viral load and treatment compliance), TB status, date of diagnosis and treatment compliance, other comorbidities, diagnostic imaging findings including size of the pericardial effusion on echocardiography, pericardiocentesis procedure and pericardial fluid analysis results, laboratory analysis, aetilogical diagnosis, basis for diagnosis. Content and face validity of the collection tool were established through expert review by the academic supervisors and by piloting the tool on a small number of eligible patient records to identify any missing or ambiguous items. To enhance reliability, the data were extracted systematically using the same tool for all records, and any ambiguous data items were discussed with supervisors to ensure consistent interpretation.

### Aetiological diagnostic criteria of pericardial effusion

Pericardial effusion aetiologies in this study were classified into predefined categories. The classification was fulfilled by the analysis of available clinical, laboratory and imaging findings. Idiopathic pericardial effusion was the final diagnosis in immunocompetent patients when no evident cause of the effusion was determined. Pericardial effusions were considered to be TB in origin when fulfilling the following criteria: (1) definite TB pericarditis based on Tubercle bacilli in stained smear or culture of pericardial fluid and/or Tubercle bacilli or caseating granulomata are on histologic examination of pericardium, (2) probable or presumed TB based on the evidence of pericarditis in a patient with TB demonstrated elsewhere in the body; and/or lymphocytic pericardial exudate with elevated adenosine deaminase (ADA) activity > 30 U/L, IFN-γ > 50 pg/mL, or lysozyme assay; and/or good response to antituberculosis treatment. Malignancy was considered a cause of pericardial effusion when it occurred in the context of a known cardiac or extracardiac malignancy or when fluid cytology included atypical or malignant cells. Human immunodeficiency virus-related pericardial effusion was determined in the absence of other identifiable causes in patients who are HIV-positive and/or with immunosuppression. The hypothyroid category was assigned to patients with elevated thyroid-stimulating hormone (TSH) > 5 mIU/L and decreased tetraiodothyronine (T4) < 12 pmol/L on laboratory findings, in the absence of other identifiable causes of pericardial effusion. Uraemic pericarditis associated with pericardial effusion was categorised when blood urea was > 15 mmol/L and creatinine > 500 µmol/L in the absence of other identifiable causes. Congestive cardiac failure patients were categorised due to a known congestive heart failure diagnosis and/or a patient with clinical features of heart failure in the absence of other identifiable aetiologies. Autoimmune diseases were considered to be aetiologies of pericardial effusion, occurring in the context of a known diagnosis of an autoimmune disease, with no other known aetiologies of pericardial effusion. Pericardial effusion cases were placed in the ‘Other’ aetiologies category after exclusion of other aetiologies. We also considered cardiac tamponade in patients who had at least three of the following clinical features: tachycardia, heart rate > 100 beats per minute, elevated jugular vein pressure > 4 cm, hypotension (systolic blood pressure < 100 mmHg), pulsus paradoxus (> 10 mmHg decreases in systolic blood pressure during inspiration) and muffled heart sounds

### Data analysis

Prior to data analysis, the dataset was examined for completeness, accuracy and consistency. The cleaned dataset was imported into IBM Statistical Package for the Social Sciences (SPSS) Statistics (Version 30) (IBM Corp., Armonk, NY, United States [US]) for analysis. Descriptive statistics were used to summarise study variables. Categorical variables (e.g. aetiology, sex, clinical presentations) were presented as frequencies and percentages, while continuous variables (e.g. age, laboratory results) were reported as means and standard deviations (s.d.) for normally distributed data or medians and interquartile ranges (IQR) for skewed data. Bivariate analysis was performed to assess associations between variables. Associations between categorical variables were evaluated using Fisher’s exact test. A *p*-value of less than 0.05 was considered statistically significant.

### Ethical considerations

Ethical approval and a consent waiver were obtained from the Research Ethics Committee of the Faculty of Medicine and Health Sciences, Walter Sisulu University (WSU HREC 419/2025) and from the Eastern Cape Department of Health (EC_202511-040). The study was conducted in accordance with the Declaration of Helsinki guidelines.

## Results

### Patient characteristics

During the study period, a total of 238 patient records were retrieved. Of these, 97 were excluded due to missing laboratory investigations and pericardial fluid analysis (some due to non-drainable effusion), thus yielding insufficient diagnostic information to determine aetiologies. A majority of this cohort was presumably diagnosed with TB pericardial effusion and on TB empirical therapy during the study period, including one with confirmed constrictive pericarditis, but had no drainable effusion. Surgical-related or post-traumatic causes of pericardial effusion, and pregnant women were excluded. The final number of records included in the study was 141. The majority of patients were aged 40 years or older, with 35.5% in the 40-year to 59-year age group and 34.0% aged 60 years or above. Younger patients aged 13–19 years accounted for a small proportion of cases (5.0%). The mean age of participants was 49.4 years (s.d. 17.0 years). Females constituted a slightly higher proportion of the study population: 74 (52.5%) females compared with males, 67 (47.5%).

Fifteen (10.6%) participants (Table 1) had other comorbid conditions not captured under predefined categories, including benign prostatic hyperplasia, ischaemic heart disease, peptic ulcer disease, epilepsy, asthma, autoimmune disorders, and selected chronic respiratory and musculoskeletal conditions. The rate of HIV was not high, and among those who were HIV-positive, the majority 55.6% was virally suppressed (Table 1).

**TABLE 1:** Characteristics and clinical findings of patients with pericardial effusion at Nelson Mandela Academic Hospital, 2022–2025.

Variable	*n*	Summary					
		%	Mean	s.d.	Median	IQR	Minimum–Maximum
**Age (years)**							
13–19	7	5.0	-	-	-	-	-
20–39	36	25.5	-	-	-	-	-
40–59	50	35.5	-	-	-	-	-
≥ 60	48	34.0	-	-	-	-	-
**Gender**							
Male	67	47.5	-	-	-	-	-
Female	74	52.5	-	-	-	-	-
**Comorbid condition**							
Hypertension	37	26.2	-	-	-	-	-
HIV infection	27	19.1	-	-	-	-	-
Diabetes mellitus	16	11.3	-	-	-	-	-
Malignancy	8	5.7	-	-	-	-	-
Chronic kidney disease	6	4.3	-	-	-	-	-
Hypothyroidism	6	4.3	-	-	-	-	-
Congestive heart failure	4	2.8	-	-	-	-	-
Chronic liver disease	1	0.7	-	-	-	-	-
Other comorbid conditions	15	10.6	-	-	-	-	-
**Viral load (copies/mL)**							
< 1000	15	55.6	-	-	-	-	-
≥ 1000	12	44.4	-	-	-	-	-
**CD4 count (cells/µL)**							
< 200	10	37.0		-	-	-	-
> 200	17	63.0		-	-	-	-
**Reported symptoms**							
Chest pain	78	55.3	-	-	-	-	-
Dyspnoea	138	97.9	-	-	-	-	-
Fatigue	119	84.4	-	-	-	-	-
Peripheral oedema	40	28.4	-	-	-	-	-
**Clinical signs on examination**							
Raised jugular venous pressure	86	61.0	-	-	-	-	-
Muffled heart sounds	130	92.2	-	-	-	-	-
Pulsus paradoxus	19	13.5	-	-	-	-	-
**Vital signs**							
**Systolic blood pressure (mmHg)**							
< 100 (*systolic hypotension)*	18	13.0	-	-	-	-	-
> 100	123	87.0	-	-	-	-	-
**Diastolic blood pressure (mmHg)**							
< 90	124	88.0	-	-	-	-	-
> 90	17	12.0	-	-	-	-	-
Heart rate (beats/min)	141	-	95.1	17.8	96.0	23.0	56–132
Respiratory rate (breaths/min)	141	-	20.8	3.3	20.0	3.0	18–32
Temperature (°C)	141	-	37.2	0.6	37.1	0.9	35.5–38.9
Oxygen saturation (%)	141	-	94.5	4.0	95.0	6.0	78–100

s.d., standard deviation; IQR, interquartile range.

### Clinical presentation

The most frequently reported symptoms, dyspnoea, fatigue, chest pain (Table 1), reflect the haemodynamic impact of pericardial effusion in these study patients rather than specific aetiologies.

Features suggestive of underlying aetiology were selectively identified. Night sweats, weight loss and cough were present in TB-related aetiology. Fever was more frequently associated with other infectious aetiologies. Weight loss and cachexia were consistent with malignancies. Patients with autoimmune diseases experience signs such as Raynaud’s phenomenon and sclerodactyly. Patients who reported constipation, cold intolerance and weight gain were diagnosed with hypothyroidism. Congestive cardiac failure associated aetiology patients had peripheral oedema, orthopnoea, paroxysmal nocturnal dyspnoea and pulmonary crackles. Pericardial friction rub was associated with uraemic causes. Signs suggestive of cardiac tamponade physiology, such as muffled heart sounds (92.2%), raised JVP (61.0%), systolic hypotension (88%) and tachycardia (43.2%) were observed.

These findings reflect the heterogeneity of clinical presentation among patients with pericardial effusion and underscore the frequent coexistence of systemic, infectious, inflammatory and cardiopulmonary features in this population.

### Imaging studies

The patients in this study had moderate-to-large pericardial effusions ranging from 13 mm to 74 mm (Table 2). Right atrial and/or right ventricular diastolic collapse was observed in all patients, a hallmark echocardiographic feature of cardiac tamponade, with no aetiological distinction in this study. Fibrous strands and a shaggy coat were predominantly present in TB- and malignant-related cases. Chest radiography results showing cardiomegaly in 81.6% of patients are consistent with moderate or large pericardial effusions in this study cohort.

**TABLE 2:** Echocardiographic and laboratory findings among patients with pericardial effusion at Nelson Mandela Academic Hospital, 2022–2025.

Variable	Summary				
	*n*	%	Median	IQR	Minimum–Maximum
**Echocardiographic features**					
Pericardial effusion size (mm) (*n* = 141)	-	-	33.00	16	13–74
Right atrial and/or right ventricular diastolic collapse (*n* = 141)	141	100.0	-	-	-
Fibrous strands (*n* = 141)	53	37.6	-	-	-
Shaggy pericardial appearance (*n* = 141)	42	29.8	-	-	-
**Radiographic features**					
Cardiomegaly (*n* = 141)	-	81.6	-	-	-
**Electrocardiography**					
Low-voltage QRS complexes (*n* = 141)	-	51.1	-	-	-
Electrical alternans (*n* = 141)	-	14.9	-	-	-
**Pericardial fluid microbiology**					
GeneXpert MTB/RIF (GXP), positive (*n* = 141)	35	24.8	-	-	-
Acid-fast bacilli (AFB) smear, positive (*n* = 141)	35	24.8	-	-	-
TB culture, positive (*n* = 141)	41	29.1	-	-	-
Cytology (malignant cells), positive (*n* = 141)	0	0.0	-	-	-
**Inflammatory markers**					
C-reactive protein (CRP) (*n* = 135)	120†	-	-	-	-
Erythrocyte sedimentation rate (ESR) (*n* = 135)	123‡	-	-	-	-
**Renal function**					
Urea (*n* = 141)	-	-	5.40	4.2 mmol/L	-
Creatinine (*n* = 141)	-	-	78.00	30 μmol/L	-
eGFR (*n* = 140)	-	-	97.00	18 mL/min/1.73 m^2^	-
**Thyroid function**					
TSH (*n* = 132)	-	-	2.94	2.3 mIU/L	-
Free T4 (*n* = 103)	-	-	13.70	5.9 pmol/L	-
**Full blood count**					
White cell count (WCC) (*n* = 141)	-	-	7.26	6.05 × 10^9^/L	-
Haemoglobin (Hb) (*n* = 141)	-	-	11.20	3.4 g/dL	-
Platelets (*n* = 141)	-	-	295.00	203 × 10^9^/L	-
Mean corpuscular volume (MCV) (*n* = 95)	-	-	88.90	11.6 fL	-
Mean corpuscular haemoglobin (MCH) (*n* = 95)	-	-	30.60	6.0 pg	-

IQR, interquartile range; eGFR, estimated glomerular filtration rate; TSH, thyroid stimulating hormone; TB, tuberculosis.

† , > 10 mg/L;

‡ , > 10 mm/hr.

### Laboratory analysis

The number of observations included in each laboratory analysis varied across tests because of missing laboratory data. Pericardial fluid microbiological analysis showed evidence of TB in a substantial proportion of patients, with GeneXpert and AFB positivity each observed in 24.8% of cases and positive TB cultures in 29.1%. No malignant cells were detected on cytological examination of pericardial fluid samples.

Inflammatory markers were generally elevated. The median C-reactive protein (CRP) was 37.0 mg/L (IQR: 89), and the median erythrocyte sedimentation rate (ESR) was 56.0 mm/hr (IQR: 73), indicating a high inflammatory burden among the patients.

Biochemical and haematological laboratory findings among patients with pericardial effusion – continuous variables are presented as median (IQR) due to non-normal distribution.

Renal function parameters indicate that most patients had preserved renal function, although a subset demonstrated reduced kidney function as reflected by the wide range of values.

Thyroid function tests showed most values within reference ranges, suggesting no widespread thyroid dysfunction in the cohort.

Haematological assessment demonstrated a median white cell count of 7.26 × 10^9^/L (IQR 6.05). The median haemoglobin concentration was 11.2 g/dL (IQR 3.4), indicating that anaemia was common in the study populations and likely associated with chronic illnesses. Platelet counts were generally elevated, with a median platelet count of 295 × 10^9^/L (IQR 203). Red cell indices showed a median mean corpuscular volume of 88.9 fL (IQR 11.6) and a median mean corpuscular haemoglobin of 30.6 pg (IQR 6.0), suggesting that the predominant anaemia pattern was normocytic and normochromic.

The laboratory profile in this study was characterised by frequent anaemia, hypalbuminaemia and inflammatory biochemical patterns, while renal and thyroid functions were generally preserved in most patients.

### Aetiologies of pericardial effusion

Based on the final diagnosis documented following retrospective review of the medical records, the aetiologies of pericardial effusion are detailed below.

#### Idiopathic pericardial effusion

Idiopathic pericardial effusion was diagnosed in cases where no underlying cause could be identified despite clinical, laboratory and imaging evaluation. This was the most common cause among HIV-negative patients (35.1%).

#### Tuberculosis pericardial effusion

**FIGURE 1:**
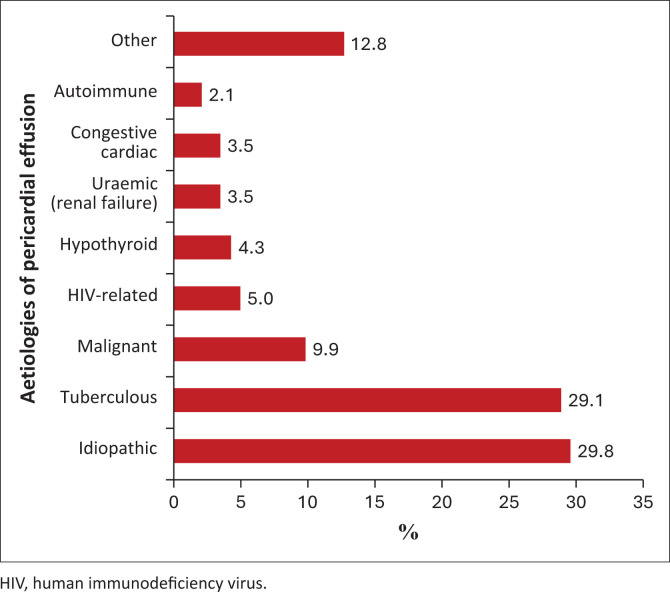
Distribution of aetiologies of pericardial effusion among medical patients at Nelson Mandela Academic Hospital.

Tuberculosis pericardial effusion was the predominant cause among HIV-positive patients, accounting for 55% of this subgroup (Figure 1, Table 3). Among the 15 patients co-infected with HIV, the majority, eight patients, demonstrated undetectable viral loads and CD4 counts > 200 cells/µL. Two additional patients had low-level viremia (< 200 copies/mL) with preserved CD4 counts. The remaining five patients were virally unsuppressed (> 1000 copies/mL) and documented as non-compliant with antiretroviral therapy. Two patients had concomitant pulmonary tuberculosis, presenting with productive cough, bilateral crackles and dyspnoea. All patients diagnosed with TB pericardial effusion had positive TB culture on pericardial fluid analysis.

**TABLE 3:** Association between the aetiology of pericardial effusion and human immunodeficiency virus status.

Aetiology category	HIV-negative		HIV-positive		Total	
	*n*	%	*n*	%	*n*	%
Tuberculosis (TB)	26	22.8	15	55.6	41	29.1
Malignant	13	11.4	1	3.7	14	9.9
Autoimmune	3	2.6	0	0.0	3	2.1
Uraemic (renal failure)	4	3.5	1	3.7	5	3.5
Hypothyroid	6	5.3	0	0.0	6	4.3
HIV-related	0	0.0	7	25.9	7	5.0
Congestive cardiac	5	4.4	0	0.0	5	3.5
Idiopathic	40	35.1	2	7.4	42	29.8
Other	17	14.9	1	3.7	18	12.8

**Total**	**114**	**100.0**	**27**	**100.0**	**141**	**100.0**

HIV, human immunodeficiency virus.

#### Malignant pericardial effusion

Malignant pericardial effusion was identified in 14 patients, with lymphoma (*n* = 6) being the most common malignancy, followed by breast cancer (*n* = 2), squamous cell carcinoma of the lung (*n* = 2), and a single case of ovarian, prostate and testicular malignancies, as well as one left atrial myxoma. One patient with diffuse large B-cell lymphoma was HIV-positive with immunosuppression (CD4 count 67 cells/µL). Weight loss and cachexia were predominant clinical features in this subgroup. The diagnosis was based on a known diagnosis of malignancy. Fluid cytology was negative for all in this subgroup; however, the CT-scan supported the diagnoses, identifying masses in the primary affected anatomical area.

#### Human immunodeficiency virus-related pericardial effusion

Human immunodeficiency virus-related pericardial effusion, in the absence of other identifiable cause, was identified in seven patients (5.0%) of the total sample of the study, representing 25.9% of the HIV-positive patients. The majority were immunosuppressed, with CD4 counts < 200 cells/µL and unsuppressed viral loads. This subset presented with symptoms in keeping with pericardial effusion, including chest pain, dyspnoea, fatigue, and three of them also demonstrated parameters that are also associated with cardiac tamponade, such as tachycardia, increased JVP, and one with systolic hypotension.

#### Hypothyroid pericardial effusion

The majority of the patients with hypothyroid pericardial effusion were female, and common symptoms were constipation, weight gain, fatigue and cold intolerance.

#### Uraemic pericardial effusion

Five patients were classified as having uraemic pericardial effusion, one of whom was HIV-positive, virally suppressed, with non-detectable viral load and with no evidence of opportunistic infection or other comorbidities. In three of these patients, pericardial friction rub was observed.

#### Congestive cardiac pericardial effusion

Congestive cardiac failure was classified as a cause of pericardial effusion. Of the five patients, four are the only ones who had pulmonary oedema in the study. The patients also presented with features classic of heart failure.

#### Autoimmune pericardial effusion

Three patients had underlying autoimmune diseases, including systemic lupus erythematosus (SLE, *n* = 1), scleroderma (*n* = 1) and mixed connective tissue disease (MCTD, *n* = 1).

#### Other aetiologies

Other aetiologies collectively accounted for a significant proportion of cases and predominantly bacterial infections, including *Enterobacter cloacae, Staphylococcus* species, *Acinetobacter baumannii, Enterococcus faecalis, Klebsiella pneumoniae* and *Bacillus cereus*, as well as one case of cytomegalovirus (CMV) infection. Fever was the most common presenting symptom in this group.

### Association of aetiology with clinical variables

Table 3 shows the distribution of pericardial effusion aetiologies stratified by HIV status. The Fisher’s exact test of *p* < 0.001 for determining the association was used. Among HIV-positive patients, TB was the predominant cause of pericardial effusion, accounting for more than half of cases (55.6%), followed by HIV-related pericardial effusion (25.9%). In contrast, HIV-negative patients most commonly had idiopathic pericardial effusion (35.1%), followed by TB (22.8%) and malignant causes (11.4%). Malignant, autoimmune, hypothyroid and congestive cardiac aetiologies were observed almost exclusively among HIV-negative patients. In summary, the distribution of aetiologies differed significantly by HIV status (Fisher’s exact test, *p* < 0.001).

Human immunodeficiency virus-related pericardial effusion occurred exclusively in HIV-positive individuals and was included for descriptive completeness; however, this category was not considered in inferential interpretation as HIV status is inherent to its definition.

**FIGURE 2:**
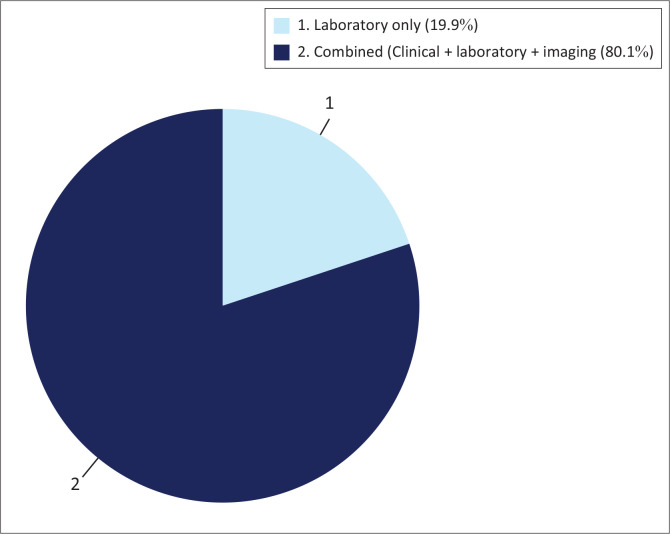
Basis of diagnosis of pericardial effusion among medical patients.

The diagnosis of pericardial effusion was most commonly established using a combined approach incorporating clinical assessment, laboratory investigations and imaging findings, accounting for 80.1% (*n* = 113) of cases (Figure 2). This indicates that the majority of diagnoses were based on multimodal assessment, reflecting comprehensive diagnostic evaluations in the study population.

## Discussion

### Socio-demographic characteristics

In this study, pericardial effusion predominantly affected middle-aged and older adults, with a mean age of 49.4 years, and the majority of patients were older than 40 years. This age distribution is consistent with the epidemiological patterns described in the literature, in which pericardial effusion is more commonly reported in adulthood due to cumulative exposure to infectious diseases, malignancies and chronic systemic conditions.^1,6,9^ Similar age profiles have been reported in African cohorts, including the Tygerberg Hospital study, where most patients with large pericardial effusions were adults.^10^

A slight female predominance was observed in this cohort. Previous studies have shown variable gender distributions, with some reporting male predominance, particularly in TB-related pericardial disease, while others report near-equal gender representation.^5,10^ The findings in this study suggest that gender differences in pericardial effusion may be influenced by local population demographics, healthcare access and comorbidity profiles rather than a true biological predisposition, as anticipated in the literature review.

### Clinical presentation

Dyspnoea was the most frequently reported symptom (97.9%), followed by fatigue (84.4%) and chest pain (55.3%). This clinical pattern is consistent with the published literature, which describes dyspnoea as the most common presenting complaint in patients with moderate to large pericardial effusions, reflecting impaired ventricular filling and reduced cardiac output.^2,15^ Chest pain, particularly pleuritic in nature, has been commonly associated with inflammatory causes of pericardial effusion, including TB and idiopathic pericarditis.^1,16^

On physical examination, muffled heart sounds (92.2%) and raised JVP (61.0%) were commonly documented, while pulsus paradoxus was infrequently recorded. This observation aligns with existing literature, which notes that although muffled heart sounds are commonly detected, pulsus paradoxus is often under-recognised or inconsistently documented in routine clinical practice, especially in resource-limited settings.^3,8^ The elevated JVP suggests a significant burden of haemodynamic instability in large pericardial effusions rather than constrictive pericarditis, which was not supported by echocardiographic findings in this cohort.

### Comorbid conditions

Hypertension was the most prevalent comorbid condition, followed by HIV infection and diabetes mellitus. This reflects the growing burden of non-communicable diseases in South Africa, alongside persistent infectious diseases.^9,12^ The coexistence of communicable and non-communicable diseases highlights the evolving epidemiological transition in developing countries.

The prevalence of HIV infection in this cohort was lower than that reported in earlier South African studies, where HIV co-infection rates exceeded 50% among patients with pericardial effusion.^10,17^ This difference may reflect improved access to antiretroviral therapy and earlier HIV diagnosis, resulting in better immune preservation. Similar trends have been reported in more recent African studies.^1,9^

### Human immunodeficiency virus-related findings

Tuberculous pericardial effusion was significantly more common among HIV-positive patients, consistent with well-established evidence linking HIV infection to extrapulmonary TB.^1,9^ However, the presence of non-tuberculous effusions among HIV-positive patients underscores the importance of comprehensive diagnostic evaluation rather than presumptive TB treatment, a key concern highlighted in the literature.^12,17^

### Echocardiographic findings

Most patients in this study had large pericardial effusions, as evidenced by a median effusion size of 33 mm (IQR 16 mm). This finding is consistent with the literature, which indicates that patients presenting to tertiary centres in developing countries often have advanced disease due to delayed presentation and referral.^5,15^ The presence of fibrous strands and a shaggy pericardial appearance on echocardiography was common. These features have been described as suggestive of tuberculous and malignant pericardial disease, particularly in high TB-burden settings.^2,5^ The findings in this study therefore support the diagnostic utility of echocardiography. The universal documentation of right atrial and/or ventricular diastolic collapse in this study likely reflects the selection of patients with clinically significant effusions requiring pericardiocentesis, rather than the prevalence of tamponade physiology in all pericardial effusions. This is consistent with descriptions in the literature that tamponade features are more common in large, rapidly accumulating effusions.^8,16^

### Chest X-ray and electrocardiogram findings

Chest radiography remains an important initial imaging modality for evaluating patients with suspected pericardial effusion, particularly in resource-limited settings.^3,4^ In this study, massive cardiomegaly was the most common chest X-ray (CXR) finding (81.6%), with the majority of patients demonstrating an enlarged cardiac silhouette. This observation is consistent with descriptions in the literature, where a globular or ‘water bottle’ cardiac silhouette is regarded as a classical radiographic feature of large pericardial effusions, usually occurring when pericardial fluid volumes exceed 200 mL – 250 mL.^8,15^

The high prevalence of cardiomegaly in this cohort correlates with the echocardiographic finding that most patients had large pericardial effusions. Similar observations have been reported in African and international studies, where cardiomegaly on chest radiography is frequently present in patients with clinically significant pericardial effusion.^2,5,10^ These findings support the role of chest X-ray as a useful screening tool for raising suspicion of pericardial effusion, although it lacks specificity. Overall, the CXR findings in this study reinforce the limited but valuable role of chest radiography in the initial assessment of suspected pericardial effusion.^3,4^

Electrocardiography is routinely performed in patients with suspected pericardial disease and may provide supportive evidence of pericardial effusion or associated pericarditis.^8,4^ In this study, low-voltage QRS complexes were the most commonly reported ECG abnormality (51.1%). This finding is well described in the literature and is attributed to the insulating effect of pericardial fluid on electrical signal transmission.^3,8^

Sinus tachycardia was frequently observed in this cohort. Similar findings have been reported in patients with large pericardial effusions and cardiac tamponade and are thought to represent a compensatory response to reduced stroke volume and systemic illness.^15,16^ Electrical alternans were infrequently observed in this study (14.9%). This aligns with the literature that describes electrical alternans as a highly specific but insensitive sign typically seen only in very large effusions with marked cardiac mobility.^3,16^ Collectively, these ECG findings mirror those reported in previous studies and highlight the supportive rather than definitive role of ECG in diagnosing pericardial effusion.^4,8^

### Laboratory findings

Inflammatory markers were markedly elevated in this cohort, with high CRP and ESR values. These findings are in keeping with inflammatory and infectious causes of pericardial effusion, particularly TB, as extensively described in the literature.^1,5^ Anaemia and hypalbuminaemia were also common, reflecting chronic inflammation, nutritional compromise and systemic disease, which are frequently reported in TB-related pericardial effusion.^9,10^

Renal and thyroid function were largely preserved in most patients, which correlates with the relatively low prevalence of uraemic and hypothyroid-related effusions. Similar findings have been reported in African studies, where metabolic causes account for a smaller proportion of cases compared to infectious and idiopathic causes.^4,5^

### Aetiological spectrum of pericardial effusion

Idiopathic and TB pericardial effusions were the most common aetiologies, each accounting for approximately one-third of cases. While TB remains a major cause, its proportion in this study is lower than previously reported South African data, where TB accounted for up to 70% of cases.^10^ This shift may reflect improved diagnostic approaches, greater recognition of alternative aetiologies and reduced reliance on empirical TB treatments.^9,12^

The substantial proportion of idiopathic effusions is consistent with global data, among which idiopathic causes account for a significant proportion of pericardial effusions despite extensive investigation.^4,11^ This finding reinforces the diagnostic challenges.

Malignancy accounted for nearly 10% of cases, aligning with previously reported African and international studies.^10,11^ This supports concerns raised in the literature regarding the rising burden of malignancy-related pericardial disease in developing countries.^12^

Aetiological classification was dependent on available investigations and clinician documentation. Despite extensive evaluation, a substantial proportion of effusions were classified as idiopathic. It is possible that some of these cases represented undiagnosed viral, autoimmune or early malignant disease.^9^

### A diagnostic approach for moderate to large pericardial effusion in a resource-constrained tuberculosis-endemic setting

Based on the results of this study, we propose a comprehensive, standardised, step-by-step approach for implementing the diagnoses of moderate to large pericardial effusion in resource-constrained and TB-endemic settings. The approach encompasses clinical assessment, echocardiography, targeted laboratory testing and pericardial fluid analysis. The diagnosis approach is as follows:

- Clinical features: Non-specific symptoms (dyspnoea, chest pain). Suspected cardiac tamponade: tachycardia (heart rate [HR] > 100 bpm), hypotension (systolic blood pressure [SBP] < 100 mmHg), raised JVP > 4 cm, muffled heart sounds and pulsus paradoxus.
- Chest X-ray: Cardiomegaly.
- Electrocardiogram: Low voltage QRS complex or electric alternans.
- Echocardiography: Confirm effusion; assess size (small or moderate or large); assess for tamponade features (Right atrial [RA] and/or right ventricular [RV] diastolic collapse).
- If moderate–large effusion (> 10 mm): Perform pericardiocentesis.
- Pericardial fluid analysis: Biochemistry, MC&S, TB culture, cytology.
- Adjunct investigations: Blood tests guided by suspicion (e.g. TB, malignancy, autoimmune, hypothyroidism, renal failure, HIV).
- Management: It is guided by aetiology.

### Strengths and limitations

This study contributes its findings to the existing body of knowledge on pericardial effusion, particularly within resource-limited and high TB and HIV prevalence settings. It addresses a significant knowledge gap by providing contemporary data on the aetiological spectrum of pericardial effusion in the Eastern Cape province, a region with limited published literature on this topic, yet burdened with TB and HIV. Given the evolving epidemiology of HIV, TB and non-communicable diseases in South Africa, the findings offer timely and locally relevant insights that can inform clinical practice and policy development. However, this study has limitations. It is a retrospective design that relies on pre-existing medical records not originally collected for research purposes. As a result, missing data and incomplete documentation were encountered, leading to the exclusion of such cases. Patients without sufficient effusion for drainage were also excluded, introducing potential selection bias. We also encountered challenges in retrieving records, as the hospital utilises only paper records. This is a single-centre study conducted at a tertiary referral hospital. Consequently, the findings may not be generalisable.

### Recommendations

It is recommended that a structured and standardised diagnostic approach be implemented for all patients presenting with moderate or large pericardial effusions, incorporating clinical assessment, echocardiography, targeted laboratory testing and pericardial fluid analysis.

Empirical treatment for TB should be avoided unless supported by clinical, radiological, microbiological or biochemical evidence, given the substantial proportion of non-tuberculous effusions identified. Clinicians should make a concerted effort to investigate aetiologies before initiating empirical therapy.

Echocardiography services should be strengthened at peripheral hospitals to facilitate earlier detection and referral of clinically significant pericardial effusions. Where feasible, access to advanced diagnostic modalities such as pericardial biopsy, GeneXpert testing of pericardial fluid and autoimmune screening should be expanded to improve diagnostic yield. Future studies including consecutive patients with pericardial effusion, including those managed empirically without pericardiocentesis, would allow for a broader comparison of aetiological patterns and strengthen the systematic diagnostic approach.

## Conclusion

This study described the aetiological spectrum and associated demographic and clinical characteristics of pericardial effusion among medical patients at NMAH in Mthatha, Eastern Cape.

Clinical presentation of pericardial effusion is poorly discriminatory for aetiology, with symptoms and signs largely reflecting the size and haemodynamic impact of the effusion rather than its underlying cause. As a result, clinical assessment alone may be insufficient.

Idiopathic pericardial effusion was the most common aetiology, accounting for 42 cases (29.8%), followed closely by tuberculous pericardial effusion in 41 patients (29.1%). A substantial proportion of effusions was attributable to non-TB conditions, including bacterial infections, malignancies, HIV-related disease, hypothyroidism, uraemia, heart failure and autoimmune conditions. This has important implications in TB-endemic regions, where empirical TB therapy is frequently initiated, risking misdiagnosis and delayed identification of alternative aetiologies. Tuberculosis pericardial effusion was significantly more common among HIV-positive patients, highlighting the persistent interaction between HIV infection and extrapulmonary TB in this region.

The diagnosis of pericardial effusion remains challenging because symptoms and signs become evident only when the effusion is large or rapidly accumulating. In resource-limited or peripheral settings, the presence of cardiomegaly on chest radiography should prompt a high index of suspicion and early referral for ultrasound evaluation to exclude pericardial effusion.

Overall, these findings support the implementation of a structured and standardised diagnostic approach that incorporates echocardiography, targeted laboratory investigations and pericardial fluid analysis where feasible, to optimise management in resource-constrained, TB-endemic settings.

This study provides contemporary, context-specific data on pericardial effusion in the Eastern Cape, helping to address the existing knowledge gap. The results have important implications for clinical practice and may inform the development of locally relevant diagnostic and management guidelines to improve outcomes for patients with pericardial effusion.
